# Supplementary material for: Seasonal Effects on Phenolic Contents and In Vitro Health-Promoting Bioactivities of Sacred Lotus (Nelumbo nucifera)
Source: Plants (Basel). 2023 Mar 24;12(7):1441. doi: 10.3390/plants12071441 (PMC10097295; doi:10.3390/plants12071441)
Supplement: Supplementary file 1 [file plants-12-01441-s001.zip › plants-2297480-supplementary.pdf]

plants

MDPI

Supplementary materials

Seasonal Effects on Phenolic Contents and In Vitro Health-promoting Bioactivities of Sacred Lotus (*Nelumbo nucifera*)

Nattira On-nom [1], Sirinapa Thangsiri [1], Woorawee Inthachat [1], Piya Temviriyanukul [1], Yuraporn Sahasakul [1], Chaowanee Chupeerach [1], Kanchana Pruesapan [2], Piyapat Trisonthi [3], Dalad Siriwan [3,*,†] and Uthaiwan Suttisansanee [1,*,†]

1 Food and Nutrition Academic and Research Cluster, Institute of Nutrition, Mahidol University, Salaya, Phuttamonthon, Nakhon Pathom 73170, Thailand; nattira.onn@mahidol.ac.th (N.O.); sirinapa.tha@mahidol.ac.th (S.T.); woorawee.int@mahidol.ac.th (W.I.); piya.tem@mahidol.ac.th (P.T.); yuraporn.sah@mahidol.ac.th (Y.S.); chaowanee.chu@mahidol.ac.th (C.C.)
2 Plant Varieties Protection Division, Department of Agriculture, Ministry of Agriculture and Cooperatives, Bangkok 10900, Thailand; kpruesapan@gmail.com (K.P.)
3 Institute of Food Research and Product Development, Kasetsart University, Chatuchak, Bangkok 10900, Thailand; piyapat.tr@ku.th (P.Tr.)
* Correspondence: dalad.s@ku.th (D.S.); uthaiwan.sut@mahidol.ac.th (U.S.)
† These authors contributed equally to this work

Supplementary Table S1:

Moisture Contents of different powdery sacred lotus parts collected at different time periods.

Copyright: © 2023 by the authors. Licensee MDPI, Basel, Switzerland. This article is an open access article distributed under the terms and conditions of the Creative Commons Attribution (CC BY) license (https://creativecommons.org/licenses/by/4.0/).

**Plant Parts**	**Time Period 1**	**Time Period 2**	**Time Period 3**
Seed embryo	9.65 ± 0.49	3.60 ± 0.02	11.03 ± 0.16
Stamen	8.37 ± 0.02	5.15 ± 0.20	1.87 ± 0.15
Old leaf	5.17 ± 0.06	4.38 ± 0.06	2.82 ± 0.22
Petal	12.12 ± 0.16	5.58 ± 0.24	2.76 ± 0.20
Flower stalk	8.15 ± 0.32	9.64 ± 0.16	6.64 ± 0.19
Leaf stalk	4.55 ± 0.35	9.24 ± 0.17	6.48 ± 0.30

Moisture contents were determined by a Halogen Moisture Analyzer (HE53 series from Mettler-Toledo AG, Greifensee, Switzerland).

Supplementary Table S2:

Color analyses of different powdery sacred lotus parts collected at different time periods.

Plant Parts	Time Period 1			Time Period 2			Time Period 3		
	**L***	**a***	**b***	**L***	**a***	**b***	**L***	**a***	**b***
Seed embryo	22.68 ± 0.01	4.55 ± 0.05	7.80 ± 0.06	41.87 ± 0.13	1.97 ± 0.01	19.99 ± 0.02	35.42 ± 0.15	(-)1.36 ± 0.06	14.23 ± 0.08
Stamen	24.18 ± 0.05	4.92 ± 0.00	12.30 ± 0.04	46.87 ± 0.3	4.95 ± 0.01	32.70 ± 0.01	33.36 ± 0.06	5.08 ± 0.02	18.32 ± 0.06
Old leaf	22.30 ± 0.04	(-)1.31 ± 0.03	10.51 ± 0.04	34.29 ± 1.76	(-)1.38 ± 0.03	12.16 ± 0.02	31.50 ± 0.03	1.11 ± 0.02	9.90 ± 0.08
Petal	20.55 ± 0.01	2.66 ± 0.01	7.24 ± 0.04	40.37 ± 0.03	1.88 ± 0.01	7.31 ± 0.04	36.74 ± 0.04	2.14 ± 0.01	7.15 ± 0.02
Flower stalk	20.56 ± 0.04	2.23 ± 0.00	7.13 ± 0.06	31.78 ± 0.03	2.08 ± 0.01	8.30 ± 0.03	36.46 ± 0.09	2.51 ± 0.03	7.69 ± 0.06
Leaf stalk	24.56 ± 0.00	2.49 ± 0.04	9.58 ± 0.04	34.42 ± 0.12	1.56 ± 0.03	12.16 ± 0.02	32.96 ± 0.04	2.86 ± 0.01	9.55 ± 0.02

Color analyses were determined using a ColorFlex EZ Spectrophotometer (Hunter Associates Laboratory, Reston, VA, USA) and expressed as CIELAB units (L* represents dark (0) to white (100) colors, a* represents green (-) to red (+) colors and b* represents blue (-) to yellow (+) colors).

Disclaimer/Publisher's Note: The statements, opinions and data contained in all publications are solely those of the individual author(s) and contributor(s) and not of MDPI and/or the editor(s). MDPI and/or the editor(s) disclaim responsibility for any injury to people or property resulting from any ideas, methods, instructions or products referred to in the content.
